# *Sox2* Ablation in the Suprachiasmatic Nucleus Perturbs Anxiety- and Depressive-like Behaviors

**DOI:** 10.3390/neurolint13040054

**Published:** 2021-10-26

**Authors:** Nicholas A. Boehler, Samuel W. Fung, Sara Hegazi, Arthur H. Cheng, Hai-Ying Mary Cheng

**Affiliations:** 1 Department of Biology, University of Toronto Mississauga, Mississauga, ON L5L 1C6, Canada; nick.boehler@mail.utoronto.ca (N.A.B.); samuel.fung@mail.utoronto.ca (S.W.F.); sara.hegazi@mail.utoronto.ca (S.H.); ahh.cheng@mail.utoronto.ca (A.H.C.); 2 Department of Cell and Systems Biology, University of Toronto, Toronto, ON M5S 3G5, Canada

**Keywords:** circadian rhythms, mood, anxiety, depression, suprachiasmatic nucleus, *Sox2*

## Abstract

Mood disorders negatively impact the lives of hundreds of millions of individuals worldwide every year, yet the precise molecular mechanisms by which they manifest remain elusive. Circadian dysregulation is one avenue by which mood disorders are thought to arise. SOX2 is a transcription factor that is highly expressed in the murine suprachiasmatic nucleus (SCN), the circadian master clock, and has been recently found to be an important regulator of *Per2*, a core component of the molecular clock. Genetic ablation of the *Sox2* gene in GABAergic neurons selectively impacts SCN neurons, as they are one of very few, if not the only, GABAergic populations that express *Sox2*. Here, we show that GABAergic-restricted ablation of *Sox2* results in anxio-depressive-like phenotypes in mice as observed in the elevated plus maze, forced swim test, tail suspension test, and sucrose preference test. We further observe a reduction in basal and/or forced swim-induced c-Fos expression, a marker of neuronal activation, in the nucleus incertus, arcuate nucleus, and dentate gyrus of *Sox2* conditional knockout (cKO) mice. Given the restricted disruption of SOX2 expression in the SCN of *Sox2* cKO mice, we propose that their mood-associated phenotypes are the consequence of a dysregulated central clock that is unable to communicate appropriately timed signals to other brain nuclei that regulate affective behaviors.

## 1. Introduction

Mood disorders, including major depression and anxiety disorders, affect an estimated 10% to 20% of individuals globally and range from temporary episodes to incapacitating conditions that chronically impact lives [1,2]. In recent years, epidemiological data indicate that the prevalence of both anxiety and depression is increasing, particularly in young adults [3,4,5]. Still, a comprehensive understanding of the molecular mechanisms that give rise to mood disorders and subsequent targets for therapeutic intervention remain elusive [6,7].

Circadian rhythms are endogenous timing systems that exist within most living organisms. Robust circadian rhythms confer an evolutionary advantage by way of enabling anticipatory physiological and behavioral adaptation to cyclical changes in the environment [8]. However, a growing body of evidence shows that abnormalities in circadian rhythms exhibit high comorbidity with mood disorders [9,10,11]. In mammals, the master circadian clock is governed by the suprachiasmatic nucleus (SCN), a bilateral structure of the anterior hypothalamus that is responsible for orchestrating daily oscillations in physiology and behavior with a period of approximately 24 h. The SCN is primarily entrained by photic cues via the retinohypothalamic tract, which conveys information regarding the day–night cycle from intrinsically photosensitive retinal ganglion cells (ipRGCs), but also by non-photic signals [12]. The temporal information is integrated and transmitted to efferent targets by various SCN output signals [13].

The molecular clock operates through a series of transcription–translation feedback loops (TTFLs) that facilitate rhythmic expression of core clock genes through negative feedback inhibition [14]. The positive arm is comprised of heterodimeric CLOCK and BMAL1 complexes that bind to E-box regulatory elements in *period* (*per1–3*) and *cryptochrome* (*cry1–2*) genes, inducing their expression. Subsequently, heterodimeric PER and CRY translocate into the nucleus and inhibit their own transcription. Upon degradation of PER and CRY proteins in the nucleus, CLOCK and BMAL1 heterodimers can begin a new 24 h cycle [14]. The oscillation of these core clock components relies on inputs from a wide array of transcription factors to maintain synchrony with geophysical time [14].

Previous findings have shown that the transcription factor SRY (sex-determining region Y)-box 2 (SOX2) is highly expressed in neurons of the adult murine SCN [15]. This contrasts with most other neuronal populations, where SOX2 expression is silenced once the cells differentiate during embryogenesis. The peculiar expression of SOX2 enabled us to generate an SCN-specific *Sox2* conditional knockout (cKO) mouse strain by crossing *Sox2^fl/fl^* mutant mice with a GABAergic-specific Cre driver strain (Vgat-cre) [16]. The resulting *Sox2* cKO mice exhibited disturbed locomotor rhythms along with reduced expression of *Per2* and various neuropeptides in the SCN, the latter likely hindering transmission of output signals to efferent targets [16]. Based on these observations, we hypothesize that ablating SOX2 expression in the SCN might disrupt the function of other brain regions that directly or indirectly receive timing signals from the SCN, including those that regulate mood and affect.

Here, we show that *Sox2* ablation in the SCN perturbs anxiety- and depressive-like behaviors in mice. *Sox2* cKO mice displayed heightened anxiety in the elevated plus maze, reduced depressive-like behavior in the forced swim and tail suspension tests, and decreased sucrose preference. Within the nucleus incertus (NI), arcuate nucleus (ARC), and dentate gyrus (DG), expression of c-Fos protein, a marker of neuronal activation, was attenuated in *Sox2* cKO mice under basal conditions and following forced swim. These findings suggest that SOX2 deficiency in SCN neurons alters the functionality of the central clock and culminates in disturbances in mood regulation.

## 2. Materials and Methods

### 2.1. Animals

All animal handling and experimental procedures were performed at the University of Toronto Mississauga (UTM) and were approved by the UTM Animal Care Committee, complying with guidelines established by the University of Toronto Animal Care Committee and the Canadian Council on Animal Care. An SCN-specific *Sox2* conditional knockout model was created by crossing *Sox2^fl/fl^*mice (JAX stock #013093) with *Vgat^cre/cre^* mice (JAX stock #028862) that were purchased from The Jackson Laboratory, as described previously [16]. These lines were crossed to obtain *Vgat^cre/+^*; *Sox2^fl/fl^* mice, hereafter referred to as *Sox2* cKO mice, as well as littermate *Sox2^fl/fl^*controls. Mice were genotyped by PCR using primers recognizing the *Sox2^flox^* allele (fwd primer: TGGAATCAGGCTGCCGAGAATCC; rev primer: TCGTTCTGGCAACAAGTGCTAAAGC) and the *Cre* transgene (fwd primer: CATTTGGGCCAGCTAAACAT; rev primer: CCCGGCAAAACAGGTAGTTA). Mice were housed separately by sex and were maintained on a fixed 12-h:12-h light–dark cycle. Food and water were provided ad libitum. Both male and female mice of approximately 2 months of age were used for all experiments.

### 2.2. Behavioral Paradigms

Behavioral experiments were conducted between Zeitgeber Time (ZT) 6 and 9. Lighting conditions were maintained at approximately 80 lux, with noise levels kept at a minimum. With the exception of the sucrose preference test, all behavioral tests were video-recorded for subsequent analysis. Three experimental cohorts, each consisting of male and female mice, were used for the four behavioral tests. Data were analyzed using a 2-way ANOVA with Tukey’s post hoc or Wilcoxon rank-sum test as appropriate, using an α set at 0.05. Open circles represent measurement from individual animals and cross marks represent outliers.

#### 2.2.1. Forced Swim Test (FST)

The forced swim test was adapted from Can et al. (2011) to quantify frequency and duration of immobility as an assessment of depressive-like behavior [17]. *Sox2* cKO mice (*n* = 6 females and 2 males) and *Sox2^fl/fl^* controls (*n* = 5 females and 2 males) were gently placed into the center of a Plexiglas container (9″ × 9″ × 18″) filled with tap water (~23–25 °C) to a depth of 12″ for a total duration of six minutes. To avoid variable acute responses to stress, only the last four minutes were quantified. Immobility was considered a lack of escape-directed behavior, defined by the movement of all four limbs in conjunction with movement. For each bout of immobility, the initial moment of immobility was recorded when the above-described condition was unmet for at least one second. The resumption of escape-directed behavior marked the end of the bout of immobility. After experimentation, mice were placed on a warming pad and dried with a soft towel before returning to their home cage. Frequency and duration of immobility were recorded for analysis.

#### 2.2.2. Tail Suspension Test (TST)

The tail suspension test was performed as previously described [18] as a second test for depressive-like behavior. Opaque, 3-sided enclosures were constructed from acrylic plastic sheets with a metal clip hanging from the closed top. Label tape was wrapped securely around the tail extremity with a 1-inch overhang to which the metal clip was attached. *Sox2* cKO mice (*n* = 2 females and 8 males) and *Sox2^fl/fl^* controls (*n* = 3 females and 8 males) were individually suspended by their tails for a duration of six minutes each. Immobility was determined by a lack of evasive behavior defined as (1) active movement of all four limbs or (2) movement of front paws in conjunction with bodily contortion. When either condition was unmet for a period of >1 s, this was recorded as the start of a bout of immobility. The resumption of either evasive behavior marked the end of a bout of immobility. Frequency and duration of immobility were quantified.

#### 2.2.3. Elevated Plus Maze (EPM)

The elevated plus maze was adapted from Walf and Frye (2007) to quantify movement behavior and open space aversion as markers for anxiety-like behavior [19]. The EPM consists of a four-armed maze with two open and two closed arms. The closed arms have opaque walls on the sides and the distal end with the roof left uncovered, while the open arms do not have any coverage and are exposed to the environment. A small box was drawn in the center to indicate the neutral middle space.

The elevated plus maze was conducted on the same cohort of mice that was used in the tail suspension test. *Sox2* cKO and *Sox2^fl/fl^*control mice were introduced in a random order to the neutral middle of the maze, initially facing the open arm. Between each subject, the maze was sanitized using 70% ethanol to remove olfactory cues. Movement into any arm required all four paws to step past the middle line into the arm; otherwise, this was considered time spent in the neutral middle space. Closed and open arm entries as well as duration spent in either arm type were quantified.

#### 2.2.4. Sucrose Preference Test (SPT)

The sucrose preference test was performed as previously described [20]. Briefly, *Sox2* cKO mice (*n* = 4 females and 4 males) and *Sox2^fl/fl^* controls (*n* = 4 females and 4 males) were singly housed and provided with two identical bottles, one containing 2% sucrose and the other water, for 48 h prior to testing. Bottle weight was measured before and after the 48 h habituation period to determine the consumption of 2% sucrose and water by each animal. On the test day, mice were deprived of fluids for 4 h (ZT 5 to ZT 9) before being presented with two identical bottles containing 2% sucrose or water for 1 h. Bottle placement during the habituation period was random, and the order was switched during testing to prevent place preference. Bottles were weighed before and after the 1 h interval. Lower sucrose preference is interpreted as anhedonia.

### 2.3. Brain Tissue Harvest and Processing

**Figure 1:**
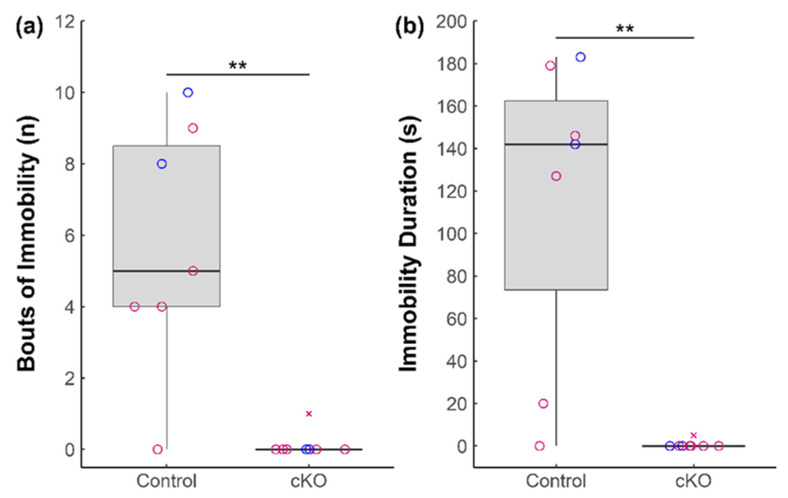
Behavioral effects of *Sox2* ablation in the SCN during the forced swim test. (**a**) Total bouts of immobility and (**b**) duration of immobility of *Sox2* cKO mice (*n* = 8) and littermate controls (*n* = 7) over the course of the forced swim test are presented. Female mice are denoted in pink and male mice are denoted in blue. Outliers are denoted by cross marks. Statistical significance from Wilcoxon rank-sum tests is marked with ** (*p* < 0.01).

Mice were killed by cervical dislocation and thick (800 μm) coronal sections from the entire rostro-caudal extent of the brain were obtained using an oscillating tissue slicer (Electron Microscopy Sciences). Tissues were fixed with 4% paraformaldehyde (PFA) in phosphate-buffered saline (PBS, pH 7.4) for 6 h at room temperature before they were transferred to 30% sucrose in PBS (pH 7.4) and stored at 4 °C. Thin (30 μm) sections were prepared from these thick coronal sections using a freezing microtome (Leica). Tissues were stored at 4 °C in 30% sucrose solution until use. Tissues were harvested from a total of 18 mice, all of which were female (6 control FST, 6 *Sox2* cKO FST, 3 control naïve, 3 *Sox2* cKO naïve). For each genotype, 3 of the 6 FST-treated mice were from the cohort for which behavioral data were analyzed and presented in Figure 1.

### 2.4. Immunohistochemistry (IHC)

Free-floating sections were washed 5 × 5 min with PBS-T (PBS with 0.1% Triton X-100), quenched with 0.3% H_2_O_2_ in PBS for 30 min, washed 5 × 5 min with PBS-T, and incubated in blocking solution for 1 h at room temperature (RT). The blocking solution contained 5% goat serum (GS) and 5% skim milk in PBS-T. Sections were incubated with rabbit anti-c-Fos antibody (1:50,000, EMD Bioscience) in 2% goat serum/5% milk in PBS-T overnight at 4 °C. The following day, sections were washed 5 × 5 min with PBS-T, incubated with biotinylated goat anti-rabbit secondary antibody (1:300, Vector Biolabs) in PBS-T with 2% GS for 2 h at RT, and washed again with PBS-T for 5 × 5 min. Sections were then incubated with avidin/biotinylated enzyme complex (ABC) solution (Vector Biolabs) for 45 min at RT before washing 5 × 5 min with PBS-T. Sections were developed with diaminobenzidine (DAB) (Vector Biolabs) according to manufacturer’s instructions. Sections were mounted on gelatin-coated microscope slides, dehydrated with ethanol and xylene, and coverslipped with Permount (Fisher Scientific, Hampton, NH, USA).

### 2.5. Image Acquisition and Analysis

Images were acquired using a Zeiss Axio Observer Z1 inverted microscope equipped with an AxioCam MRm Rev.3 monochromatic digital camera (Zeiss) using the Zen 2010 software (Zeiss). Identical settings were used for imaging all samples. All quantitative image analyses were performed on ImageJ. All brain regions that exhibited c-Fos immunoreactivity were initially analyzed for mean IHC intensity. Each area of interest (AOI), chosen by cross-referencing area size and shape with the Allen Brain Atlas, was outlined with the polygon selection tool, and the average optical density was obtained using the “measure” function. Background staining was measured in a non-immunoreactive region adjacent to the AOI and subtracted from the immunoreactive intensity of the AOI. Only brain regions that demonstrated a significant difference in mean c-Fos IHC intensity between *Sox2* cKO and control mice were selected for further analysis of c-Fos (+) cell counts. To quantify c-Fos (+) cell numbers, manual counts were conducted within the AOI using cell_counter.jar as an added plugin. Cell counts were quantified by number of c-Fos (+) cells/AOI area to obtain density of c-Fos (+) cells. Two sections were taken from each mouse for density quantification of c-Fos (+) cells, and these were averaged together. The nucleus incertus was missing in 1 of 3 control naïve, 2 of 6 control FST, and 1 of 6 *Sox2* cKO FST samples due to damage of this small brain region from the initial vibratome sectioning.

### 2.6. Statistical Analysis

Statistical analyses were performed with Wilcoxon rank-sum tests, and 2-way ANOVAs with Tukey’s post hoc as appropriate on RStudio 3.4.2 with α set to 0.05.

## 3. Results

### 3.1. Ablation of Sox2 in the SCN Severely Alters Anxiety-like and Depressive-like Behaviors in Mice

To determine whether ablation of *Sox2* in the SCN impacts anxiety- and depressive-like behaviors, we subjected *Sox2* cKO mice and *Sox2^fl/fl^* controls to four behavioral paradigms established to assess a rodent’s affective state. In the forced swim test, immobility is interpreted as depressive-like behavior in response to a stressful yet inescapable situation. *Sox2* cKO mice demonstrated a significant and substantial reduction both in the number of bouts of immobility (Figure 1a), as well as duration of immobility in comparison to control mice (Figure 1b).

**Figure 2:**
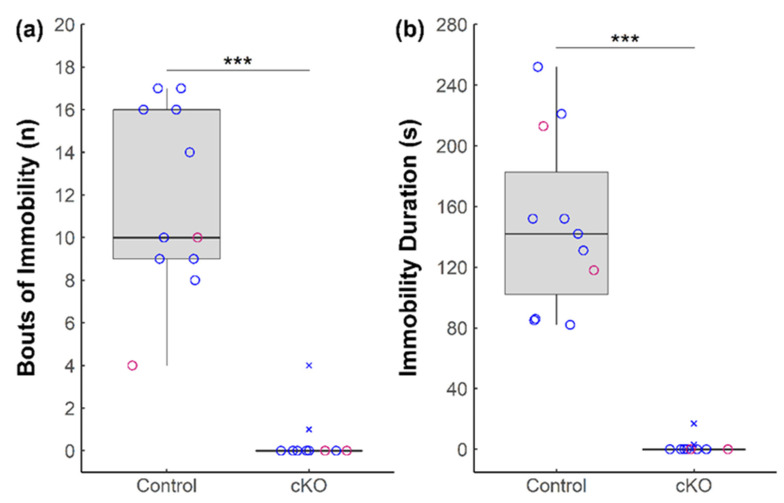
Behavioral effects of *Sox2* ablation in the SCN during the tail suspension test. (**a**) Total bouts of immobility and (**b**) duration of immobility of *Sox2* cKO mice (*n* = 10) and littermate controls (*n* = 11) during the tail suspension test. Female mice are denoted in pink and male mice are denoted in blue. Outliers are denoted by cross marks. Statistical significance from Wilcoxon rank-sum tests is marked with *** (*p* < 0.001).

Similar to the FST, immobility in the tail suspension test is interpreted as an index of depression. During the TST, *Sox2* cKO mice exhibited a reduction in the number of bouts of immobility (Figure 2a) and duration of immobility (Figure 2b) compared to controls. Notably, the majority (8 out of 10) of *Sox2* cKO mice were mobile for the entire six minutes of the TST. 

**Figure 3:**
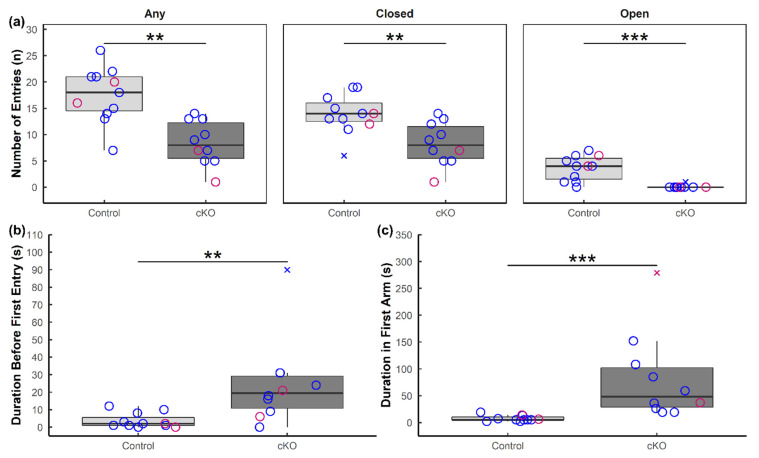
Anxiety-like behavior in *Sox2* cKO mice observed during the elevated plus maze. (**a**) Number of entries of *Sox2* cKO mice (*n* = 10) and littermate controls (*n* = 11) into any arm of the EPM, only closed arms, and only open arms. (**b**) Amount of time before the subject first left the neutral middle and entered any arm of the EPM. (**c**) Duration of time the subject spent in the first arm of the EPM. Female mice are denoted in pink and male mice are denoted in blue. Outliers are denoted by cross marks. Statistical significance from Wilcoxon rank-sum tests is marked with ** (*p* < 0.01) or *** (*p* < 0.001).

Next, we used the elevated plus maze to investigate behaviors that are classically interpreted as anxiety. These include an increased aversion to the open space of the EPM as well as reduced explorative behavior. Compared to controls, *Sox2* cKO mice exhibited fewer instances of explorative behavior, as indicated by the reduced frequency of entry into any arm, closed or open (Figure 3a, left). *Sox2* cKO mice made significantly fewer entries into the closed arms relative to control animals (Figure 3a, middle) and, more remarkably, they almost never entered the open arms (Figure 3a, right). Moreover, following placement in the maze, *Sox2* cKO mice remained in the neutral middle for a significantly longer period before selecting a first arm to explore (Figure 3b), and remained longer in the first arm prior to leaving and entering another part of the maze (Figure 3c).

**Figure 4:**
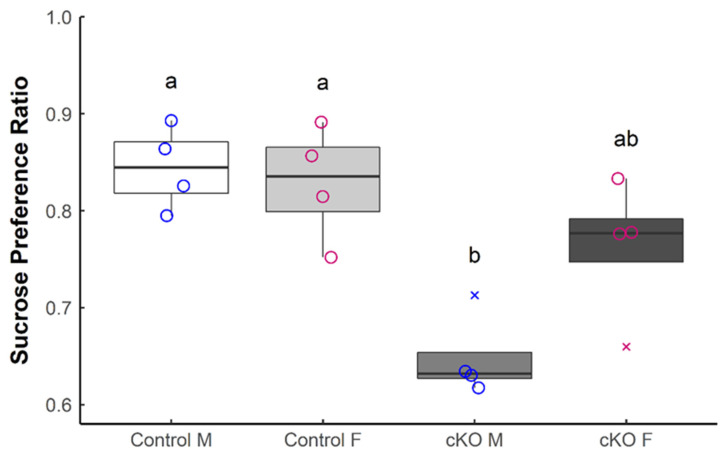
*Sox2* ablation reduces sucrose preference in male mice. The sucrose preference ratio during the test phase was determined for *Sox2* cKO mice (*n* = 8) and littermate controls (*n* = 8). Males (M) and females (F) are considered separately. Female mice are denoted in pink and male mice are denoted in blue. Outliers are denoted by cross marks. Statistical significance from 2-way ANOVA with Tukey’s Post hoc test is represented by letter differences (*p* < 0.05).

Lastly, we assessed sucrose preference of *Sox2* cKO mice and littermate controls. The sucrose preference test evaluates hedonic drive for a natural reward, in this case a sweet-tasting solution. Reduced consumption of the sucrose solution is classically interpreted as an index of anhedonia, or lack of interest in rewarding stimuli, although this interpretation is controversial ([21,22,23,24]). There was a significant reduction in sucrose preference in the *Sox2* cKO mice relative to controls during the test phase (Figure 4). Notably, sex-based analysis revealed that *Sox2* cKO males exhibited reduced preference for the sucrose solution compared to control animals of both sexes, while *Sox2* cKO females were not statistically different from control groups of either sex (Figure 4). Collectively, our data demonstrate that abolishing SOX2 expression in GABAergic neurons of the SCN perturbs behaviors that are associated with anxiety, depression, and motivation (or anhedonia) in mice.

### 3.2. Altered Neuronal Activation in Sox2 cKO Mice

To investigate the neural underpinnings of the affective phenotypes of *Sox2* cKO mice, we asked whether neuronal activity patterns in select brain regions were altered in these animals. Using c-Fos as a marker of neuronal activity, we examined its immunoreactivity (IR) throughout the rostrocaudal extent of the whole brain in unstressed (basal) and stressed mice 90 min after forced swim. Based on mean c-Fos-IR intensity, only three brain regions exhibited a significant difference in expression between genotypes: these are the nucleus incertus, the dentate gyrus, and the arcuate nucleus of the hypothalamus (data not shown). The numbers of c-Fos-IR cells in these brain regions were subsequently quantified to confirm genotype- and treatment-specific effects.

**Figure 5:**
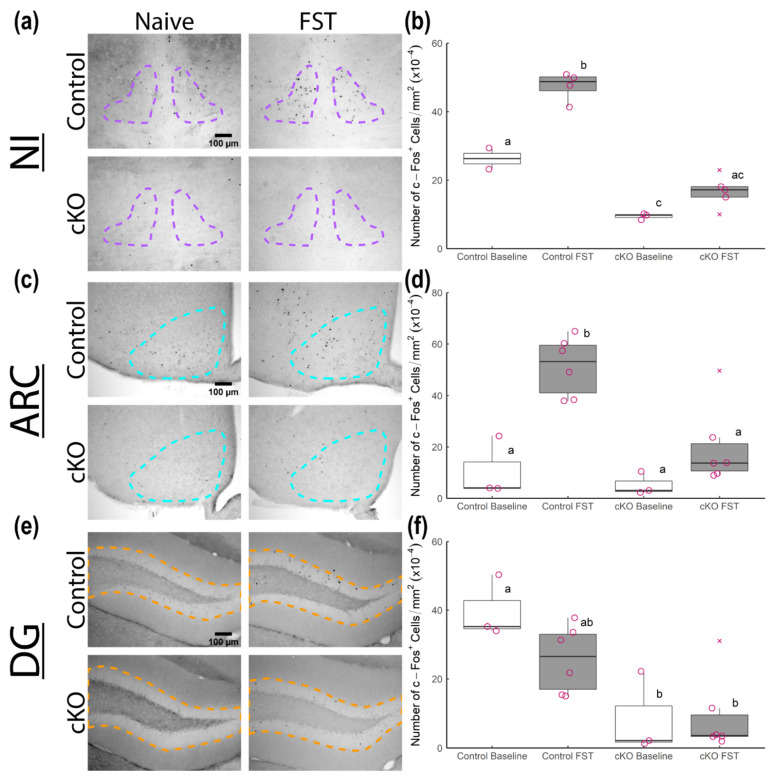
c-Fos induction in various brain regions of *Sox2* cKO mice and littermate controls following forced swim. (**a**,**c**,**e**) Representative micrographs of anti-c-Fos stained coronal sections with outlines for the nucleus incertus (NI), arcuate hypothalamic nucleus (ARC) and dentate gyrus (DG), respectively. The left-most micrographs are from naïve mice while the right-most micrographs are from mice 90 min after forced swim test (FST). (**b**,**d**,**f**) Density of c-Fos-immunoreactive nuclei in each brain region of naïve *Sox2* cKO mice (*n* = 3), naïve littermate controls (*n* = 2 for NI, *n* = 3 for ARC/DG), FST-stressed *Sox2* cKO mice (*n* = 5 for NI, *n* = 6 for ARC/DG), and FST-stressed littermate controls (*n* = 4 for NI, *n* = 6 for ARC/DG). All mice were female, with outliers represented by cross marks. Statistical significance from 2-way ANOVA with Tukey’s post hoc test is represented by letter differences (*p* < 0.05).

The nucleus incertus has been functionally implicated in arousal and responses to stress [25,26]. There were significantly fewer c-Fos-IR nuclei in the NI of *Sox2* cKO mice compared to controls under both basal conditions and following forced swim (Figure 5a,b). Importantly, forced swim triggered a significant increase in the number of c-Fos-IR cells in the NI of control mice but not of *Sox2* cKO mice (Figure 5a,b).

The arcuate nucleus has garnered attention as an important player in the regulation of corticosterone secretion [27]. A recent study demonstrated that the reciprocal connections between the ARC and the SCN are essential for corticosterone rhythms [28]. Under basal conditions, the number of c-Fos-IR nuclei in the ARC was similar between control and *Sox2* cKO mice (Figure 5c,d). Following forced swim, control mice exhibited a significant elevation in the abundance of c-Fos-IR cells in the ARC (Figure 5c,d). However, this induction was not observed in *Sox2* cKO mice (Figure 5c,d).

The dentate gyrus is one of the two major sites of adult neurogenesis in the murine brain, and as such has been linked to the regulation of cognition and mood [29]. There was no significant change in the number of c-Fos-IR cells in the DG following forced swim in either control or *Sox2* cKO mice (Figure 5e,f). However, *Sox2* cKO mice had far fewer c-Fos-IR cells in the DG relative to control animals at the basal level (Figure 5e,f). Collectively, our data suggest that loss of SOX2 expression in the SCN reduces neuronal activation in the NI, ARC, and DG under basal and/or stressful conditions.

## 4. Discussion

Here, we demonstrate that selective ablation of *Sox2* in the SCN alters anxiety- and depressive-like behaviors in mice. *Sox2* cKO mice exhibited less explorative behavior in the EPM, venturing into new arms less frequently and spending less total time in open arms than control animals. During the FST, all *Sox2* cKO mice except for one were mobile for the entire duration of the test, in contrast with control mice which exhibited bouts of immobility. Similarly, during the TST, *Sox2* cKO mice exhibited more escape-directed behavior and fewer bouts of immobility. The SPT suggests that male *Sox2* cKO mice have a lower sucrose preference than control animals of either sex. Lastly, our c-Fos data suggest that basal neuronal activity in the NI and DG may be reduced in *Sox2* cKO mice, and that forced swim may not trigger the induction of neuronal activation in the NI and ARC of these mutant animals to the same extent as it does in controls.

There exists a growing body of evidence linking disruption in circadian rhythms to a variety of mood disorders, including depression, bipolar disorder, and anxiety [30,31]. Numerous studies have shown a clear link between shift work and both depression and anxiety [32,33,34,35]. In addition, there is ample evidence from rodent studies demonstrating that circadian disruption coincides with phenotypes reminiscent of mood disorders. For example, forced desynchrony of the SCN through exposure to 22 h light-dark cycles increases depressive-like behaviors in rats [36]. In mice, SCN-specific ablation of *bmal1* exacerbates both depressive- and anxiety-like behaviors [37], whereas global deletion of *cry1* and *cry2* preferentially increases anxiety-associated behaviors [38]. Concomitant knockdown of *per1* and *per2* in the nucleus accumbens of mice also elevates anxiety-like behavior [39].

Results from our *Sox2* cKO mice are in line with studies that positively correlate circadian disturbances with an increase in anxiety-like behaviors [37,38,39]. Using the EPM as a test of anxiety, we find that *Sox2* cKO mice were much less inclined to explore the maze, as indicated by the reduced frequency of entry into any arm, and they almost never entered the open arms. After spending an extended period of time in the neutral middle, most *Sox2* cKO mice chose to alternate between the two closed arms, avoiding the open arms altogether. This aversion to exploring the maze and entering the open arms is unlikely to be the result of motor impairment or hypoactivity, as evidenced by their increased mobility in the FST. Furthermore, although freezing behavior was not quantified, we noted a propensity for *Sox2* cKO mice to remain immobile while spending time in the closed arms (data not shown). These findings illustrate the importance of SOX2 expression in the SCN in the negative modulation of anxiety-related behaviors. Whether this effect is dependent on circadian phase is unclear, as the EPM was performed at only one time of the day, in the middle of the animal’s rest phase (ZT 6–ZT 9). Another outstanding issue is whether the circadian perturbations that we have previously reported in these animals [16] lead to sleep disturbances that in turn exacerbate anxiety-like behaviors. Interestingly, a recent study showed that chronic sleep deprivation in mice induced anxiety-like phenotypes in the EPM [40].

To study depression, we used two popular paradigms of behavioral despair, the FST and the TST. In both tests, the subject is confronted with a situation that is highly stressful yet inescapable [41]. After a period of time spent struggling to escape, the subject will eventually abandon its efforts and become immobile, a behavior that is classically interpreted as reflecting a state of depression. Unexpectedly, *Sox2* cKO mice displayed almost no immobile behavior in the FST and the TST, a finding that is contrary to other studies, suggesting that manipulations disruptive to circadian rhythms enhance depressive-like behaviors in rodents [36,37]. We do not believe that the behavior of *Sox2* cKO mice in the FST and TST merely reflects a potential hyperactive phenotype [16], which is based solely on previous data suggesting heightened daytime wheel-running activity; otherwise, the effects of hyperactivity should also be reflected in the EPM test, which is not the case. The anthropomorphic interpretation of a rodent’s immobility in the FST as a sign of depression has come under increasing challenge, encouraging us to re-evaluate our results through a slightly different lens. For instance, de Kloet and Molendijk (2016) suggested that mobile and immobile behaviors in the FST represent two different coping mechanisms—active versus passive—to a stressful situation, rather than indicating a non-depressed or depressed state, respectively [42]. An alternative explanation offered by Anyan and Amir (2018) views anxiety as being the root cause of mobile behaviors aimed at providing escape from the stressful situation. Conversely, they argue that immobility does not necessarily reflect a depressed state [43]. Based on Anyan and Amir’s reinterpretation of the FST, we propose that reduced immobility in the FST and TST is the consequence of a heightened state of anxiety in *Sox2* cKO mice, consistent with their behavior in the EPM. Our interpretation does not preclude the possibility that severe anxiety in these animals prevents them from switching to a passive coping mechanism—immobility—in dealing with the stressful situation, as suggested by De Kloet and Molendijk.

In humans, anhedonia is one of the core symptoms of major depressive disorder, which is often comorbid with general anxiety disorder [44]. To infer anhedonia in rodents, the sucrose preference test is most commonly employed, even though the test fundamentally measures the behavior of an animal towards a rewarding stimulus and is not a direct measure of its ability to experience pleasure. On this cautionary note, we found that *Sox2* cKO mice have a reduced preference for sucrose compared to control animals. While this effect of *Sox2* ablation is statistically significant, the phenotype of *Sox2* cKO mice is less striking on the SPT than it is on the other behavioral tests, in particular the FST and TST where a floor effect was observed in the mutants. Furthermore, in contrast to the other tests, the reduction in sucrose preference appears to be restricted to *Sox2* cKO male mice. No sex differences were detected in the other behavioral tests, due in part to the small sample size of one sex in each of these tests but also to the aforementioned floor effect displayed by the mutant animals in the FST and TST. Given the small sample sizes in our behavioral assays, the potential for sexually dimorphic phenotypes warrants further investigation.

As a first step towards identifying the neural mechanisms underlying the mood-associated phenotypes of *Sox2* cKO mice, we undertook a stereological examination of c-Fos expression under basal conditions and following forced swim. Striking differences in c-Fos immunoreactivity were found in the nucleus incertus, arcuate nucleus, and dentate gyrus of *Sox2* cKO mice compared to controls, although this does not preclude the possibility that there may be more subtle changes in other brain regions. Our c-Fos data suggest that the absence of SOX2 in the SCN leads to a reduction in the basal activation of NI and DG neurons. More importantly, induction of neuronal activation in the NI and ARC in response to forced swim, which has been observed in previous studies [45,46], is blunted or abolished in *Sox2* cKO mice. In other words, the NI and ARC appear to be refractory to the effects of forced swim and possibly other emotional stressors when SOX2 is absent in the central circadian pacemaker. This is particularly interesting in light of the fact that *Sox2* cKO mice persist in their escape-directed behavior for almost the entire duration of the forced swim.

Whether the NI and ARC are the anatomical substrates driving the mood-associated phenotypes of *Sox2* cKO mice remains to be determined, although there is evidence to suggest their involvement. For instance, NI neurons respond to physiological stressors and the stress hormone, corticotropin-releasing factor (CRF), and are a major source of the neuropeptide, relaxin-3 (RLN3), in the rodent brain [26,47]. Both anxiogenic and anxiolytic effects of RLN3 signaling from the NI have been demonstrated depending on the behavioral paradigm used and whether exogenous or endogenous RLN3 is manipulated [48,49,50]. The NI and RLN3 have been suggested to play a modulatory role in arousal, stress, and affect [51]. The ARC is better known for its involvement in energy balance and the stress response, the latter through its role in regulating corticosterone secretion [27]. However, signaling from the ARC can influence the affective state of rodents. For example, inhibition of pro-opiomelanocortin (POMC)-expressing ARC neurons has been shown to reduce the duration of immobility in the FST and TST in mice previously subjected to chronic restraint stress [52]. Another study revealed that the anxiolytic effects of fasting were diminished when the activity of agouti-related protein (Agrp)/neuropeptide Y (NPY) neurons in the ARC was inhibited [53].

A key, unanswered question is how the absence of SOX2 in the SCN is able to impact the function or activity of other brain regions to modulate mood-related behaviors. We previously showed that not only were molecular rhythms damped in the SCN of *Sox2* cKO mice, but that many of the important neuropeptides synthesized by the SCN are strongly downregulated at the gene and/or protein level [16,54]. These observations suggest that both cellular timekeeping within SCN neurons and the ability of the SCN to convey temporal information to other brain regions may be impaired. Although there is no evidence so far that the NI receives direct projections from the SCN, the ARC is directly innervated by SCN neurons that synthesize the neuropeptides, arginine vasopressin (AVP), vasoactive intestinal peptide (VIP), and prokineticin 2 (PK2) [55], whose expression are all drastically reduced in *Sox2*-deficient SCN. Interestingly, PK2 signaling has been implicated in mood disorders in humans and anxio-depressive-like behaviors in mice [56,57]. It is possible that signals sent by the SCN to direct—or even indirect—targets can influence the basal activity of the latter and their responsiveness to other stimuli. Alternatively, SCN outputs may modify the nature and/or strength of other incoming signals that these brain regions receive. Future studies should determine whether the effects of *Sox2* ablation in the SCN on mood-related behaviors and neuronal activation in the NI, ARC and other brain regions are time-of-day-dependent.
